# From ERAS to FRAS: the significant innovation in perioperative care

**DOI:** 10.1097/MS9.0000000000004899

**Published:** 2026-04-02

**Authors:** Jing Chen, Yan Wang, Lei Wang, Ruhong Shi, Huimin Zhou, Chen Chen, Zehui Shun, Xiaohuang Tu, Congbing Yan

**Affiliations:** a Department of Gastrointestinal Surgery, Shanghai Fourth People’s Hospital Affiliated to Tongji University, Shanghai, China; b Department of Nursing, Shanghai Fourth People’s Hospital Affiliated to Tongji University, Shanghai, China; c Department of Pain, Shanghai Fourth People’s Hospital Affiliated to Tongji University, Shanghai, China

*Dear Editor,*

Enhanced Recovery After Surgery (ERAS) has transformed perioperative management; however, its limitations in individualization, pain control, and psychological support reveal a critical gap addressed by nursing-led innovations. Our team developed the Fast Recovery After Surgery (FRAS) concept, which emphasizes nursing-driven personalized care, seamless coordination throughout the perioperative process, and patient-centered interventions. This article summarizes the current status and shortcomings of ERAS, introduces our nursing-focused FRAS research, and discusses its implications for enhancing clinical practice.

## The nursing perspective on ERAS: current status and limitations

The ERAS protocol, a multimodal perioperative care framework, has demonstrably reduced hospital stays and complication rates across various surgical specialties[1]. Despite its successes, the standardized nature of ERAS protocols can inadvertently marginalize the pivotal role of nursing in managing patient heterogeneity, potentially compromising the full potential for recovery.

### Insufficient individualization in nursing practice

The standardized, “one-size-fits-all” nature of many ERAS pathways often does not adequately incorporate nuanced nursing assessments of critical patient characteristics, such as pain tolerance, baseline mobility, and psychological status[2]. This lack of integration limits the personalization of care. For instance, while early mobilization is a core ERAS component, elderly patients with cognitive impairment may not benefit without nurse-led, tailored education. Similarly, blanket analgesic regimens may be ineffective or harmful for opioid-sensitive individuals, a risk that could be mitigated by proactive nursing assessment and intervention.

### Inadequate pain management: a nursing gap

Although multimodal analgesia within ERAS (e.g., combining opioids and NSAIDs) is a cornerstone for pain control, its implementation often lacks a structured mechanism for nurse-led individualization. Nurses, as the frontline caregivers who continuously monitor patients, are uniquely positioned to titrate analgesic regimens based on real-time feedback. However, existing ERAS protocols seldom formally empower them with the autonomy to make these critical adjustments, creating a gap between protocol and patient-centered execution[3].

### Lagging nutritional support: nursing’s untapped potential

While ERAS rightly emphasizes early postoperative feeding, it often overlooks the critical importance of nursing-led nutritional support across the entire perioperative continuum. This includes preoperative screening for malnutrition risk and intraoperative advocacy for metabolic stabilization. Without proactive preoperative nutritional assessments by nurses and their subsequent coordination with clinical dietitians, high-risk patients (e.g., those with cancer) may face suboptimal nutritional intake, potentially leading to complications such as delayed wound healing[4].

### Marginalized psychological care: nursing’s core responsibility

Psychological well-being is a key determinant of recovery, yet ERAS protocols typically lack formally integrated, nursing-led psychological interventions. Nurses, by virtue of their sustained patient contact, are uniquely qualified to identify and address anxiety, depression, and fears that directly impair adherence to rehabilitation protocols. Despite this, their role within the ERAS framework is often circumscribed, relegating them to task-oriented functions rather than leveraging their full capacity for therapeutic emotional support[5].

## FRAS: a nursing-driven innovation for perioperative care

**Figure 1.**
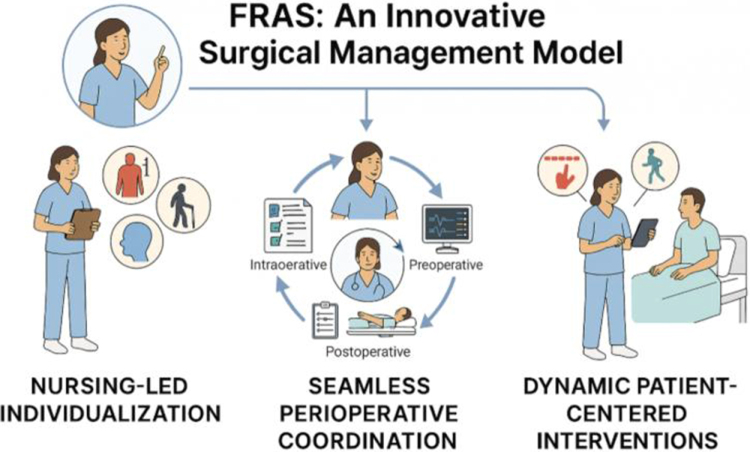
FRAS overview diagram.

In direct response to the identified limitations of standardized ERAS protocols, our team developed the FRAS model. FRAS represents a paradigm shift towards a nurse-led care framework, fundamentally grounded in three synergistic core principles: nursing-led individualization, seamless care coordination across the entire perioperative journey, and dynamic patient-centered interventions[6]. The core principles include the following (Fig. 1).

### Nursing-led individualization

This principle moves beyond the “one-size-fits-all” approach by mandating that comprehensive nursing assessments – utilizing validated tools for pain, functional mobility (e.g., Timed Up and Go test), and psychological status – form the dynamic and foundational basis for all subsequent care plans. Care is therefore not merely administered but is continuously tailored and adapted to the individual’s evolving needs and responses.

### Seamless perioperative coordination

FRAS repositions the nurse as the central coordinator of care, ensuring continuity from the preoperative through to the postoperative phase. This encompasses preoperative education and preparation, vigilant intraoperative monitoring and advocacy, and proactive postoperative management including structured follow-up, thereby creating a cohesive and uninterrupted recovery trajectory.

### Dynamic patient-centered interventions

Central to FRAS is the formal empowerment of nurses with the autonomy to make real-time, evidence-informed adjustments to care. This is activated in direct response to continuous patient feedback regarding symptoms such as pain levels and activity tolerance. This principle transforms the nurse’s role from a protocol implementer to an autonomous clinical decision-maker, thereby directly aligning care with patient-reported outcomes and enhancing patient agency.

## Nursing implementation of FRAS: clinical evidence

A retrospective cohort study was conducted involving 263 surgical patients at our institution between January 2022 and December 2023. The cohort included 16 patients who received the novel FRAS protocol (nurse-led) and 247 historical controls managed under standard ERAS protocols. Despite the disparity in group sizes, reflective of the phased implementation of FRAS, statistical comparisons using appropriate tests (e.g., Student’s *t*-test, Mann–Whitney *U* test, Chi-square test) revealed significant improvements across multiple short-term outcomes in the FRAS group, as detailed in Table 1.

**Table 1:** Comparison of short-term outcomes between FRAS and ERAS.

Outcome	FRAS (*n* = 16)	ERAS (*n* = 247)
Mean VAS score (POD0–3)	0.2	0.6
Mean ambulation time	0.00833 days(0.2 h)	1 day
Mean oral feeding time	0.075 days(1.8 h)	1 day
Mean hospital stay	1.08 days(26 h)	3.7 days
Mean medical cost	¥51.9K	¥68.1K
All-cause complications	0%	6.7%
Unplanned reoperation	0%	0.4%
Unplanned readmission	0%	0.8%

POD, postoperative days; VAS, Visual Analog Scale.

### Superior pain control: nursing-led individualization

Patients under the FRAS protocol demonstrated significantly lower postoperative pain, as evidenced by a lower mean VAS score (0.2 vs. 0.6, *P* < 0.01). This superior pain control is attributable to a structured, nurse-driven protocol implemented across all phases. Commencing preoperatively, nurses conducted individualized pain tolerance assessments and educated patients on non-opioid alternatives. Intraoperatively, they proactively monitored for signs of inadequate analgesia, facilitating real-time adjustments. Postoperatively, nurses employed an analgesic “ladder,” tailoring regimens – such as combining non-opioid medications with psychological support for anxious patients – based on continuous assessment.

### Faster rehabilitation: nursing-led mobilization

Rehabilitation was markedly accelerated in the FRAS group, with a significantly earlier mean ambulation time (0.2 h vs 24 h [1 day], *P* < 0.01). This was driven by a systematic, nurse-led mobilization strategy. Preoperative mobility baselines were established using standardized tools like the Timed Up and Go test, enabling tailored education. Intraoperatively, collaboration with anesthesiologists minimized sedation to promote early awakening. Postoperatively, nurses consistently used positive reinforcement and quantified progress feedback (e.g., “You have successfully walked 10 steps”) to motivate and achieve mobilization goals.

### Reduced hospital stay: nursing-led coordination

The FRAS model significantly reduced the average hospital stay (26 h vs 3.7 days, *P* < 0.01), a testament to the efficacy of seamless, nurse-coordinated care across the perioperative continuum. This involved preoperative coordination with dietitians for nutritional optimization, intraoperative vigilance regarding fluid balance, and proactive postoperative management using structured discharge criteria (e.g., a Postoperative Discharge Scoring System) coupled with condition-specific patient education (e.g., meticulous wound care instructions for diabetic patients) to ensure safe and timely discharge.

### Enhanced safety: nursing-led risk prevention

The FRAS group recorded no all-cause complications (0%), a rate significantly lower than that observed in the ERAS group (6.7%, *P* < 0.05). This enhanced safety profile is attributed to a proactive, nurse-driven safety culture embedded within the model. This encompassed preoperative comorbidity screening and collaboration with the multidisciplinary team (MDT) for preemptive optimization, intraoperative monitoring and early warning of potential issues like hypotension, and rigorous postoperative surveillance through structured rounding to identify early signs of complications such as surgical site infection.

## The value of FRAS for nursing practice: empowerment and impact

The significance of FRAS extends beyond being an incremental improvement to ERAS. It represents a fundamental paradigm shift in perioperative care. This nurse-led model creates substantial value by catalyzing the following transformative changes in nursing practice:

### Professional empowerment and role expansion

FRAS strategically empowers nurses by granting them the autonomy and accountability for clinical decision-making, enabling real-time care adjustments in response to patient feedback. This transition from a task-oriented executor of standardized protocols to an autonomous, evidence-based practitioner is pivotal for realizing the full potential of professional nursing.

### Direct and measurable impact on patient outcomes

Our findings provide compelling evidence that nursing-led individualization is not merely an adjunct but a primary driver of superior clinical outcomes. The FRAS model directly translates into quantifiable benefits, including significantly reduced pain scores, accelerated functional recovery, shortened length of stay, and a markedly enhanced patient safety profile.

### Fostering genuine interprofessional collaboration

The model intrinsically positions nurses as the central coordinators and nexus of the MDT. This necessitates and fosters proactive, structured collaboration with surgeons, anesthesiologists, dietitians, and other providers. Consequently, FRAS moves beyond siloed care, cultivating a truly integrated, team-based approach that is integral to achieving optimized patient outcomes.

## Future directions: nursing-led FRAS research

The promising initial findings of this study necessitate further rigorous investigation to establish FRAS as a robust, evidence-based standard in perioperative nursing. Future research should be strategically directed along several key avenues. First, large-scale, multi-center randomized controlled trials are imperative to validate the efficacy of FRAS across diverse surgical populations, such as oncology and orthopedics, thereby confirming its generalizability. Concurrently, there is a critical need to develop and psychometrically validate standardized nursing assessment tools and intervention protocols (e.g., for pain, mobility, and psychological distress) to facilitate consistent implementation and fidelity in clinical practice. Finally, expanding the outcome measures to include long-term indicators – such as 30-day readmission rates, patient-reported satisfaction, and health-related quality of life at 90 days or beyond – will provide a more comprehensive understanding of the model’s sustained impact. Together, these research efforts will consolidate the evidence base, refine the FRAS framework, and ultimately guide its successful integration into standard care to maximize patient recovery.

## Conclusion

In conclusion, while the ERAS framework has undeniably advanced the field of perioperative care, its inherent limitations underscore the indispensable role of nursing in achieving optimal patient outcomes. The FRAS model, introduced and empirically supported by our clinical data, establishes that a care paradigm centered on nursing-led individualization, seamless perioperative coordination, and dynamic patient-centered interventions can profoundly enhance recovery trajectories. As the nursing profession increasingly drives innovation in this domain, FRAS emerges not merely as an alternative, but as a promising new standard capable of delivering more rapid, safe, and holistically compassionate patient care.

## Data Availability

The authors have nothing to report.
